# pH-Dependent Antibacterial Activity of Glycolic Acid: Implications for Anti-Acne Formulations

**DOI:** 10.1038/s41598-020-64545-9

**Published:** 2020-05-04

**Authors:** Elba R. Valle-González, Joshua A. Jackman, Bo Kyeong Yoon, Natalia Mokrzecka, Nam-Joon Cho

**Affiliations:** 1 School of Materials Science and Engineering, Nanyang Technological University, 50 Nanyang Avenue 639798, Singapore, Singapore; 2 School of Chemical Engineering, Sungkyunkwan University, Suwon, 16419 Republic of Korea

**Keywords:** Antimicrobials, Bacteria, Pathogens, Microbiology techniques

## Abstract

Glycolic acid is the smallest alpha hydroxy acid and widely used for skincare applications, including to treat acne vulgaris. Oftentimes, high concentrations of glycolic acid (~20–50 vol%) are incorporated into chemical peels to reduce acne-related inflammation while there is an outstanding need to determine to what extent glycolic acid can potently inhibit *Cutibacterium acnes* (formerly known as *Propionibacterium acnes*), which is a Gram-positive bacterium implicated in acne pathogenesis. Herein, we report that glycolic acid exhibits pH-dependent antibacterial activity against *C. acnes* and mechanistic studies identified that the nonionic form of glycolic acid is more active than the anionic form. The degree of antibacterial activity, including minimum bactericidal concentration (MBC), of glycolic acid was evaluated in the pH range of 3 to 4.5, and the greatest potency was observed at pH 3. In light of skincare formulation needs, we selected the pH 3.5 condition for further testing and determined that glycolic acid kills *C. acnes* cells by disrupting bacterial cell membranes. While most conventional treatments involve high concentrations of glycolic acid (>20%), our findings support the potential of developing anti-acne formulations with glycolic acid concentrations as low as 0.2% and with pH conditions that are suitable for over-the-counter applications.

## Introduction

Acne vulgaris is a chronic inflammatory disease of hair follicles that causes cosmetically unfavorable lesions on the skin surface, which makes it a leading dermatological problem worldwide^1,2^. The origin of acne vulgaris is multifaceted and involves a few key steps^3^. First, the excessive production of oily secretions from hair follicles can cause proliferation of pathogenic *Cutibacterium acnes* (*Propionibacterium acnes*) bacterial strains on the skin surface and in the follicles^4,5^. In some cases, this bacterial overgrowth and accompanying changes in skin microflora can induce hyperkeratinization and inflammation, which triggers the formation of skin lesions^6^. There are many strategies to treat acne vulgaris and one promising approach involves using antibiotics to inhibit *C. acnes* on the skin surface^7^. However, antibiotic treatments can have drawbacks such as skin irritation and the emergence of antibiotic-resistant *C. acnes* strains^8–10^. These issues have led to the exploration of natural antibacterial solutions^11^ such as membrane-disruptive antimicrobial fatty acids that can inhibit *C. acnes* while posing lower risks for resistance development^12–14^. Such strategies have led to the growing rise in topical dermocosmetics to treat acne vulgaris^15,16^.

Within this scope, glycolic acid – an important alpha hydroxy acid – merits attention because it is one of the most widely used natural compounds in the skincare industry and is readily extracted from fruit juices and sugar cane^17^. Glycolic acid is a key component of aqueous solutions used in chemical peeling procedures, in which case the outermost layer of the skin surface is exfoliated in order to rejuvenate the skin by reducing scarring and inflammation^18^. In patients with acne vulgaris, glycolic acid treatment can lead to significant reductions in the number of skin lesions^19^. Typically, high glycolic acid concentrations (>30 vol%) are used for skin exfoliation and pore unclogging while lower concentrations (<15 vol%) are used to prevent pore occlusion^20^. Thus, chemical peeling procedures based on glycolic acid are currently used to treat acne vulgaris as an adjuvant treatment^21,22^. There is also some evidence that glycolic acid might reduce hyperkeratinization as well^23^. Interestingly, Takenaka *et al*. reported that 30–35 vol% glycolic acid exhibits antibacterial activity against *C. acnes* and can decrease *C. acnes* concentrations on the cheeks of acne vulgaris patients in a human clinical trial^24^. Notably, the chemical peel used in that study contained 35 vol% glycolic acid at pH 1.2, which is not suitable for over-the-counter topical use^25^. Current guidance supported by the US Food and Drug Administration advises that glycolic acid formulations in dermocosmetic products be within the range of ≤ 10 vol% glycolic acid concentration and formulation pH ≥ 3.5. Therefore, it would be advantageous to further explore the antibacterial properties of glycolic acid in order to devise more broadly useful treatment strategies that are suitable for over-the-counter application usage.

Herein, we investigated the concentration-dependent antibacterial activity of glycolic acid against *C. acnes* in different pH conditions and identified that glycolic acid inhibits *C. acnes* at >100-fold lower concentrations than previously reported (down to ~0.2% glycolic acid at pH 3.5). We tested the pH-dependent range of antibacterial activity to inhibit bacterial cell viability, followed by determining the minimum bactericidal concentration (MBC) values of glycolic acid at specific pH conditions. It was identified that glycolic acid potently inhibits *C. acnes* in the pH range of 3–4.5. Within this pH range, glycolic acid had greater potency at lower pH when more glycolic acid molecules were in the nonionic form. Mechanistic studies further supported that glycolic acid is bactericidal and disrupts *C. acnes* cell membrane integrity. Taken together, our findings support that glycolic acid inhibits *C. acnes* bacteria and is thus a promising agent to treat acnes vulgaris, especially since its mechanism of action could potentially offer a higher barrier to resistance development as compared to currently used antibiotics.

## Results and Discussion

### Antibacterial potency as a function of solution pH

**Figure 1:**
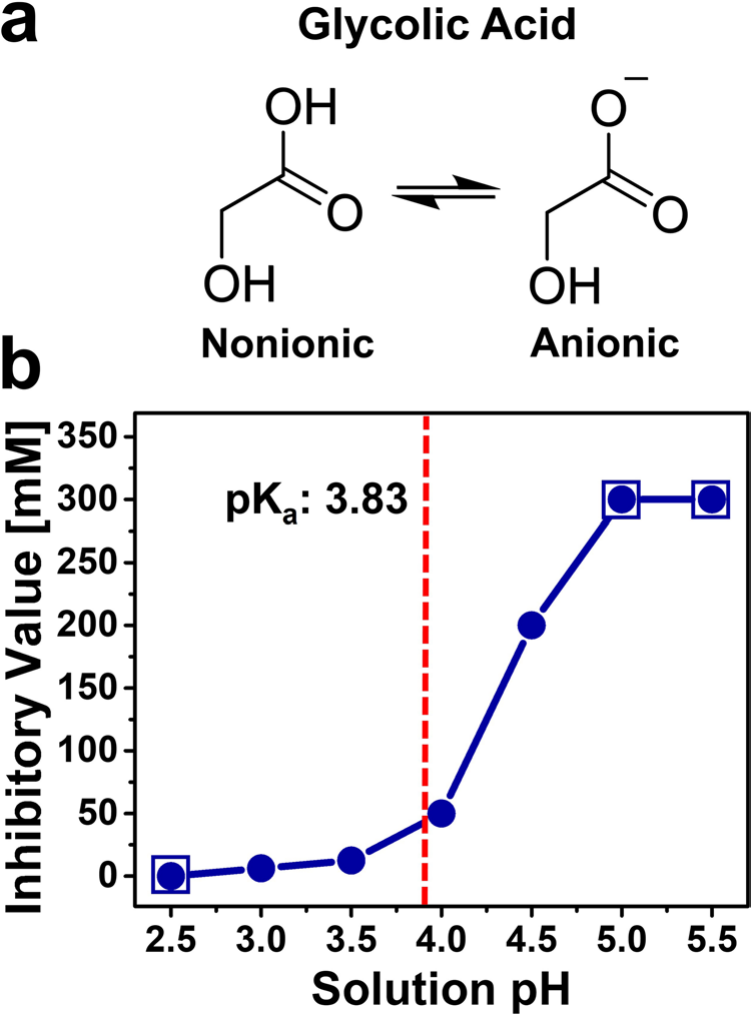
Evaluation of glycolic acid as an antibacterial agent to inhibit *C. acnes* viability. (**a**) Molecular structure of glycolic acid in the nonionic (protonated) and anionic (deprotonated) states. The equilibrium ratio of glycolic acid molecules in the two states depends on the pH condition. **(b)** Experimentally determined lowest concentration of glycolic acid to fully inhibit *C. acnes* viability in different pH conditions. The *C. acnes* cell concentration was 1 × 10^6^ CFU mL^−1^ and cell suspensions were incubated in glycolic acid solutions at different pH conditions for 1 hr before agar plating to determine if glycolic acid treatment inhibited cell viability. Each data point is representative of three independent experiments. The boxed-in circles indicate pH conditions where an inhibitory concentration was not recorded for one of two reasons: the pH condition itself during the incubation step caused loss of *C. acnes* viability (pH 2.5) or glycolic acid was inactive (*i.e*., not antibacterial) within the test range up to 200 mM (pH 5.0 and 5.5). The dashed vertical line represents the pK_a_ value of glycolic acid, which is around pH 3.83.

Glycolic acid is the smallest alpha hydroxy acid and consists of a carboxylic acid functional group along with a hydroxyl functional group at the neighboring α-carbon position^26^ (Fig. 1a). Only the carboxylic acid group is ionizable in biologically relevant pH conditions and glycolic acid exists in an equilibrium between two molecular states: (1) nonionic when the carboxylic acid is protonated at low pH conditions and (2) anionic when the carboxylic acid is deprotonated at high pH conditions. The acid disassociation constant, pK_a_, is defined as the pH value at which half of the glycolic acid molecules are nonionic and the other half are anionic. It has been reported that the pK_a_ value of glycolic acid is around pH 3.83 (ref. ^27^).

Therefore, we first evaluated the antibacterial activity of glycolic acid in different pH conditions. *C. acnes* suspensions were incubated for 1 hr in two-fold-diluted sets of glycolic acid solutions (200 mM to 1.6 mM) and each set had been prepared at a specific pH value between 2.5 and 5.5, followed by agar plating to determine the lowest glycolic acid concentration at which no bacterial growth was visible due to antibacterial activity at the incubated pH condition (Fig. 1b). At pH 2.5, the solution was too acidic to support subsequent *C. acnes* viability so no inhibitory concentration was recorded for glycolic acid in that case. By contrast, *C. acnes* was still viable after incubation at pH 3.0 and the corresponding inhibitory concentration of glycolic acid at which bacterial growth was fully inhibited was 6.3 mM. At higher pH values, *C. acnes* remained viable after incubation and glycolic acid exhibited pH-dependent antibacterial activity. The corresponding inhibitory concentrations of glycolic acid at pH 3.5, 4.0, and 4.5 were 12.5 mM, 50 mM, and 200 mM, respectively. At higher pH values of 5.0 and 5.5, glycolic acid did not exhibit antibacterial activity in the test range up to 200 mM. Together, the data support that glycolic acid exhibits greater antibacterial potency at lower pH values.

By application of the Henderson-Hasselbalch equation, these findings further support that glycolic acid has more potent antibacterial properties when it is present in its nonionic form. At pH values of 3.0 and 3.5, more than 87% and 68% of glycolic acid molecules are in the nonionic form, respectively. By contrast, at pH values of 4.0 and 4.5, less than 41% and 18% of glycolic acid molecules are in the nonionic form, respectively.

### Characterization of bactericidal activity

**Figure 2:**
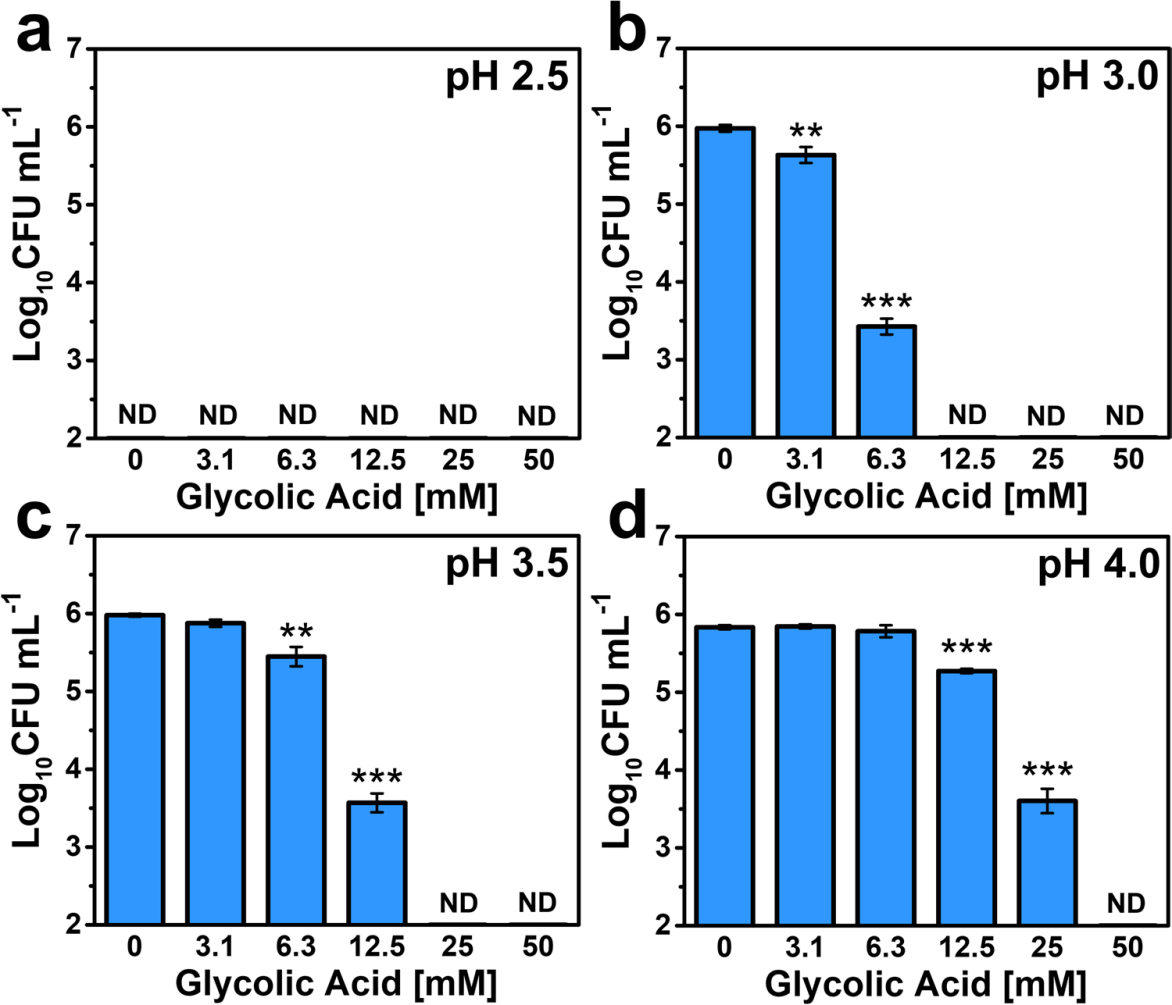
pH-dependent bactericidal activity of glycolic acid against *C. acnes*. The pH-dependent reduction in viable *C. acnes* cell concentration is plotted as a function of glycolic acid concentration. The data correspond to **(a)** pH 2.5, **(b)** pH 3.0, **(c)** pH 3.5, and **(d)** pH 4.0. The *C. acnes* cell concentration was 1 × 10^6^ CFU mL^−1^. The recorded MBC values are 12.5 mM, 25 mM, and 50 mM at pH 3.0, 3.5, and 4.0, respectively, and correspond to the lowest glycolic acid concentration at which no bacterial cell viability was detected (reported as ND, not determined). *C. acnes* cells were not viable after incubation in the pH 2.5 condition, whereas viability was maintained after incubation in the other test pH conditions as indicated by the 0 mM glycolic acid control data for each case. Mean ± standard deviation values are reported from *n* = 3 experiments.

We next conducted colony-forming unit (CFU) enumeration assay experiments in order to evaluate the concentration range at which glycolic acid kills *C. acnes* (by at least 99.99%) in different pH conditions, including determining the minimum bactericidal concentration (MBC) values. At pH 2.5, the solution was too acidic to promote *C. acnes* viability and thus no MBC value was recorded (Fig. 2a). At pH 3.0, there was *C. acnes* viability with a viable cell concentration around 1 × 10^6^ CFU·mL^−1^ and glycolic acid exhibited concentration-dependent bactericidal activity (Fig. 2b). Treatment with 3.1 and 6.3 mM glycolic acid significantly reduced the viable cell concentration to around 7 × 10^5^ CFU·mL^−1^ and 4 × 10^3^ CFU·mL^−1^, respectively. The MBC value recorded at pH 3.0 was 12.5 mM.

At pH 3.5, the bactericidal activity of glycolic acid was still significant but less potent (Fig. 2c). There was a negligible reduction in cell viability upon treatment with 3.1 mM glycolic acid, whereas treatment with 6.3 and 12.5 mM glycolic acid significantly reduced the viable cell concentration from around 1 × 10^6^ CFU·mL^−1^ to 7 × 10^5^ CFU·mL^−1^ and 4 × 10^3^ CFU·mL^−1^, respectively. The MBC value recorded at pH 3.5 was 25 mM. At pH 4.0, glycolic acid still exhibited bactericidal activity but it was even less potent (Fig. 2d). Treatment with 3.1 and 6.3 mM glycolic acid led to negligible reductions in cell viability. On the other hand, treatment with 12.5 and 25 mM glycolic acid significantly reduced the viable cell concentration from around 1 × 10^6^ CFU·mL^−1^ to 4 × 10^5^ CFU·mL^−1^ and 6 × 10^3^ CFU·mL^−1^, respectively. The MBC value recorded at pH 4.0 was 50 mM. Together, the MBC data corresponded well with the aforementioned results and support that glycolic acid exhibits pH-dependent antibacterial activity against *C. acnes*.

### Microscopic observation of bacterial cell killing

**Figure 3:**
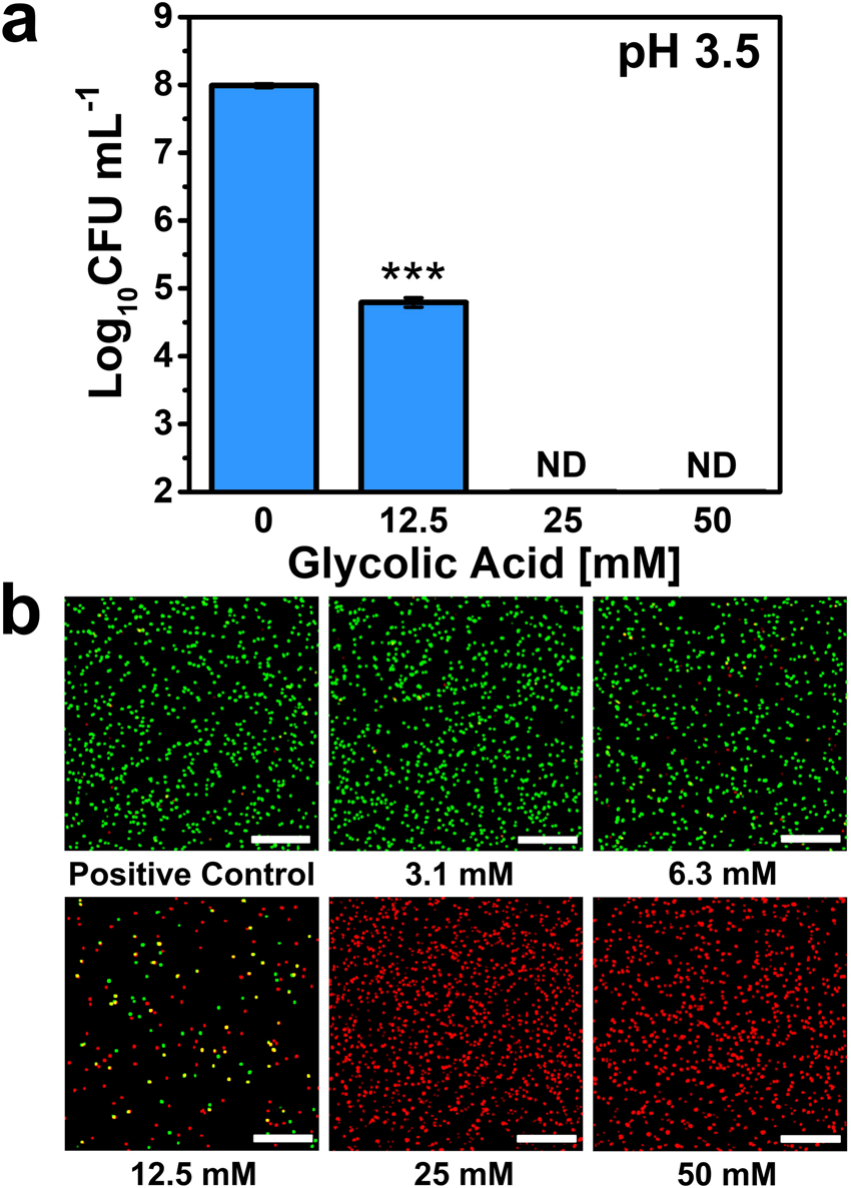
Effect of glycolic acid treatment on *C. acnes* cell viability. (**a**) Effect of glycolic acid concentration-dependent treatment on viable *C. acnes* cell concentration at pH 3.5. The *C. acnes* cell concentration was 1 × 10^8^ CFU mL^−1^. The recorded MBC value was 25 mM, and corresponds to the lowest glycolic acid concentration at which no bacterial cell viability was detected (reported as ND, not determined). Mean ± standard deviation values are reported from *n* = 3 experiments. **(b)** Live-dead assay evaluation of glycolic acid-treated *C. acnes* cells at different glycolic acid concentrations by confocal laser scanning microscopy (CLSM) imaging. Green and red colors indicate live and dead *C. acnes* cells, respectively. Images are representative of three experiments. Scale bars: 100 μm.

To confirm cell killing at pH 3.5, confocal microscopy imaging was performed to distinguish live and dead *C. acnes* cells upon treatment with glycolic acid. A two-fluorophore staining approach was used, whereby the SYTO 9 dye (green color) can translocate across all bacterial cell membranes while the PI dye (red color) can only permeate the cell membranes of dead bacterial cells with compromised membrane integrity^28^. Using a CFU enumeration assay, we first confirmed that glycolic acid decreases *C. acnes* cell viability at a higher cell density of 1 × 10^8^ CFU mL^−1^ (100-fold higher than the antibacterial testing conditions described above and necessary for cell imaging purposes) (Fig. 3a).

The basic operating principles of the microscopy imaging protocol were also confirmed using a positive control (live; green color) sample consisting of untreated *C. acnes* cells incubated in pH 3.5 solution (Fig. 3b). The data support that, under the testing conditions, *C. acnes* remained alive in the pH 3.5 condition in the absence of glycolic acid. We proceeded to investigate the concentration-dependent killing of *C. acnes* cells in the concentration range of 3.1 to 50 mM glycolic acid. With increasing glycolic acid concentration, the fraction of live cells decreased and complete killing was observed from 25 mM glycolic acid upward, as indicated by the red color stain of all visible cells. This finding is consistent with the MBC data and led us to further investigate the effects of glycolic acid treatment on bacterial cell membrane permeability.

### Evaluation of membrane permeabilization

**Figure 4:**
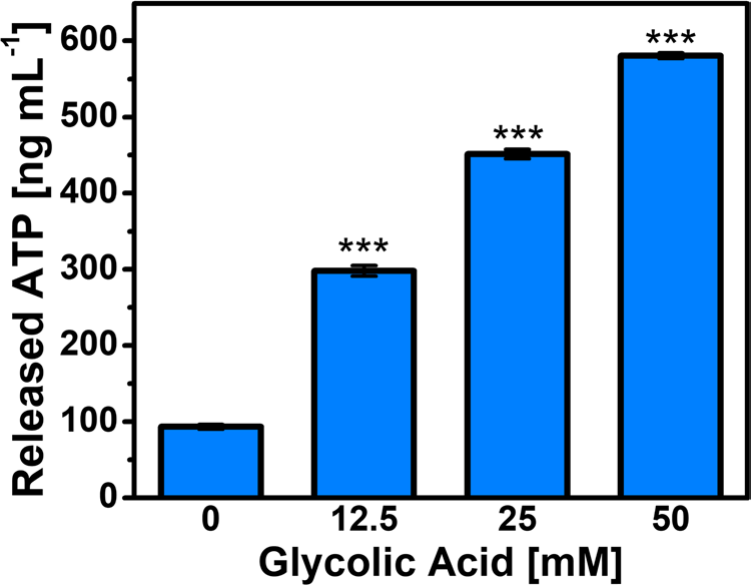
Effect of glycolic acid treatment on bacterial cell membrane integrity. The concentration of extracellular ATP released from *C. acnes* cells was measured by bioluminescence assay. The *C. acnes* cell concentration was 2 × 10^8^ CFU mL^−1^. Mean ± standard deviation values are reported from *n* = 3 experiments.

We tested the effects of glycolic acid treatment on *C. acnes* cell membrane permeability at pH 3.5 by monitoring the release of adenosine triphosphate (ATP), which is a sensitive marker of membrane damage^29,30^. *C. acnes* cells were incubated with different concentrations of glycolic acid and the amount of ATP released was determined by a bioluminescence readout. We tested glycolic acid concentrations in the range of 12.5 to 50 mM along with appropriate controls and the data are presented in Fig. 4. In untreated *C. acnes* cells at pH 3.5, the mean ATP concentration was only 93 ng mL^−1^ while there was a concentration-dependent increase in released ATP with mean values of 298, 451, and 581 ng mL^−1^ corresponding to treatment with 12.5, 25, and 50 mM glycolic acid, respectively. Noting that the MBC value was 25 mM in this case, the data indicate that complete killing of *C. acnes* cells occurs when there is a nearly-five-fold increase in membrane permeability. This finding supports that glycolic acid is bactericidal and its mechanism of action involves membrane disruption. Furthermore, Pérez-Isidoro *et al*. have reported that the nonionic, protonated form of glycolic acid has greater rates of membrane translocation than the ionic, deprotonated form, which helps to explain why glycolic acid exhibits pH-dependent antibacterial activity involving a membrane-disruptive mechanism^31^.

### Implications for anti-acne formulation development

As discussed in the Introduction, glycolic acid is widely used in chemical peels for skincare applications. Notably, the glycolic acid concentrations used in chemical peels are typically high and in the range of 20–50 vol%. While there have been several studies discussing the benefits of glycolic acid chemical peels for overall acne treatment to repair scars and reduce inflammation, to our knowledge, there has been only one previous report^24^ that discusses how glycolic acid can directly inhibit *C. acnes*. In that study, a high concentration of 30 vol% glycolic acid was used and rapid killing was noted in pH 1.5 and 3.5 solutions while less efficient killing was observed at pH 5.5. Building on this past work, our findings in this study advance mechanistic insight and demonstrate that far lower concentrations of glycolic acid exhibit high bactericidal activity against *C. acnes*. We discovered that glycolic acid concentrations as low as 0.1, 0.2, and 0.4 vol% completely kill *C. acnes* at pH 3.0, 3.5, and 4.0, respectively. Importantly, these data reveal that glycolic acid is 150-times more potent at pH 3.5 than previously discussed in the literature and open the door to creating mild anti-acne formulations with low-concentration glycolic acid samples for expanded over-the-counter usage.

## Conclusions

In this work, we have demonstrated that glycolic acid exhibits potent antibacterial activity against *C. acnes*, especially in acidic pH environments around pH 3 to 4.5 that are suitable for over-the-counter formulation development. While glycolic acid is typically used at relatively high concentrations in chemical peel treatments, our findings reveal that glycolic acid is inhibitory at far lower concentrations and devising formulation strategies in this suitable pH range could lead to more targeted and longer-duration use of glycolic acid to treat acne vulgaris, especially to reduce *C. acnes* levels while also taking advantage of its broader anti-inflammatory functions. From a mechanistic perspective, there are two key molecular-level insights resulting from this work: (1) the antibacterial potency of glycolic acid against *C. acnes* depends on the solution pH. The data indicate that glycolic acid is more active when it mainly exists in the nonionic (protonated) form, which is consistent with a greater tendency of this form to partition into phospholipid membranes as compared to the anionic (deprotonated) form; and (2) glycolic acid damages the integrity of *C. acnes* cell membranes as part of its bactericidal activity. Considering the longstanding challenges of drug-resistant *C. acnes* strains emerging to conventional antibiotics, the membrane-disruptive mechanism of action of glycolic acid is particularly significant because there is a documented high barrier to bacterial resistance developing against other classes of natural, membrane-active antibacterial drugs such as free fatty acids and similar possibilities might hold for glycolic acid as well. In summary, glycolic acid is a promising antibacterial agent that potently inhibits *C. acnes* in acidic pH environments suitable for over-the-counter formulations and its membrane-disruptive bactericidal mechanism of action could be useful for improving acne vulgaris treatment strategies.

## Materials and Methods

### Materials

Glycolic acid and sodium hydroxide were procured from Sigma-Aldrich (St. Louis, MO). Tryptic soy broth (TSB) and the BD GasPak EZ Incubation Container System, including sachets, were obtained from Becton Dickinson (Franklin Lakes, NJ). Defibrinated sheep blood was supplied by Thermo Fisher Scientific (Waltham, MA). Tryptic soy agar plates with 5% defibrinated sheep blood were acquired from Hardy Diagnostics (Santa Maria, CA). Phosphate-buffered saline (PBS) was procured from Gibco (Carlsbad, CA). The Live/Dead BacLight Bacterial Viability Kit was obtained from Invitrogen/Molecular Probes (Carlsbad, CA). The ATP Bioluminescence Assay Kit HS II was procured from Roche (Mannheim, Germany). All solutions were prepared with Milli-Q-treated deionized water (>18 MΩ ∙ cm resistivity) (MilliporeSigma, Burlington, MA).

### Glycolic acid preparation

Stock solutions of 200 mM glycolic acid were prepared in PBS. The initial solution pH was around 2.3 and the pH was adjusted by adding 3 M NaOH, followed by stirring and pH monitoring using an electronic pH meter (Accumet AB15, Thermo Fisher Scientific; Waltham, MA). The procedure was repeated until reaching the desired pH value. Before experiment, glycolic acid samples were heated at 55 °C for 30 min, and then cooled down before measurements were conducted at room temperature. The solution pH was rechecked immediately before experiment.

### Bacterial cell culture

A quality control strain of *C. acnes* (ATCC 11827, American Type Culture Collection, Manassas, VA) was cultured in Tryptic soy broth with 5% defibrinated sheep blood for 48 hrs under anaerobic conditions using a Gas-Pak (80% N_2_, 13% CO_2_, 7% H_2_) at 37 °C. The bacterial suspension was then re-inoculated in fresh Tryptic soy broth with 5% defibrinated sheep blood and incubated under the same anaerobic conditions for an additional 24 hrs. The bacterial cells were next harvested by centrifugation at 1485 × *g* for 10 min, washed thrice with PBS, and re-suspended in the same buffer. The OD_600_ value was measured and the appropriate dilution was made to reach a value of ~0.35 (mid-exponential growth phase)^32^. This value corresponds to a density of 2 × 10^8^ CFU mL^−1^, as confirmed by colony-forming unit (CFU) enumeration in control experiments. The bacteria suspension was diluted with PBS to a density of 2 × 10^6^ CFU mL^−1^ for testing purposes.

### Antibacterial testing

The antibacterial activity of glycolic acid samples was evaluated by incubating *C. acnes* suspensions in glycolic acid solutions, followed by agar plating to determine resulting *C. acnes* viability based on colony growth. At different pH values, glycolic acid samples were tested in the concentration range of 200 mM to 1.6 mM in a two-fold dilution series in a 96-well plate format. The glycolic acid samples were incubated with *C. acnes* at a concentration of 1 × 10^6^ CFU mL^−1^ under anaerobic conditions for 1 hr at 37 °C before spotting onto tryptic soy agar plates supplemented with 5% defibrinated sheep blood. The streaked plates were then incubated under anaerobic conditions at 37 °C for 4 days, after which the presence of bacterial colonies in each test group was evaluated. The reported inhibitory concentrations of glycolic acid in different pH conditions were determined by identifying the lowest concentration of glycolic acid that completely inhibited the visible growth of bacterial colonies upon agar plating. All experiments were performed in triplicate, including positive controls (bacteria incubated without glycolic acid at test pH conditions) and negative controls (solution without bacteria at test pH conditions).

### Minimum bactericidal concentration (MBC) testing

Glycolic acid samples were prepared at different pH values in the concentration range of 200 mM to 1.6 mM and incubated with *C. acnes* at a concentration of 1 × 10^6^ CFU mL^−1^. The samples were cultured under anaerobic conditions for 1 hr at 37 °C, and then each sample was diluted in a 10-fold series and streaked onto tryptic soy agar plates supplemented with 5% defibrinated sheep blood. The streaked plates were incubated under anaerobic conditions at 37 °C for 4 days, and the CFU density of each sample was next determined. The effect of glycolic acid concentration on cell viability was determined at each tested pH condition along with the corresponding MBC value, which was defined as the lowest concentration of glycolic acid that reduced bacterial cell viability by at least 99.99%. All experiments were performed in triplicate, including positive controls (bacteria incubated without glycolic acid at test pH conditions) and negative controls (solution without bacteria at test pH conditions).

### Live/Dead bacterial cell staining

Glycolic acid samples were prepared at pH 3.5 in the concentration range of 50 mM to 3.1 mM and then added to *C. acnes* suspensions at a final concentration of 1 × 10^8^ CFU mL^−1^. The samples were incubated under anaerobic conditions for 1 hr at 37 °C and then stained with fluorescent dyes by using the Live/Dead BacLight Bacterial Viability Kit (Molecular Probes, Invitrogen, Carlsbad, CA) according to the manufacturer’s protocol. The stained bacterial samples were then observed using an LSM 710 confocal laser scanning microscope (Zeiss, Oberkochen, Germany) along with appropriate negative and positive controls. Live and dead bacterial cells could be visualized by green and red stains, respectively.

### ATP release measurements

*C. acnes* suspensions were diluted to an OD_600_ value of ~0.5 that corresponds to a density of 4 × 10^8^ CFU mL^−1^. Glycolic acid samples were prepared at pH 3.5 in the concentration range of 50 mM to 12.5 mM and then added to *C. acnes* suspensions at a final concentration of 2 × 10^8^ CFU mL^−1^. The samples were incubated under anaerobic conditions for 1 hr at 37 °C, followed by titrating the samples with 3 M NaOH to increase the pH value to ~7.5, which is suitable for bioluminescence measurements. Then, the samples were analyzed by the ATP Bioluminescence Assay Kit HS II (Roche), which is based on the luciferase-catalyzed oxidation of luciferin that emits light. The relative amount of bioluminescence was measured by using a Cytation 5 cell imaging multi–mode microplate reader (BioTek, Winooski, VT). The bioluminescent signals were converted into ATP concentrations based on a standard curvature.

### Statistical analysis

Data were analyzed with GraphPad Prism software (San Diego, CA) and compared by one-way analysis of variance (ANOVA) with Dunnett’s multiple comparisons test (versus untreated control, indicated by *) in Figs. 2 and 4 or by the unpaired student’s t-test in Fig. 3. The statistical significance was computed in terms of a multiplicity-adjusted P values, and *P* < 0.05, *P* < 0.01, and *P* < 0.001 indicate the levels of statistical significance (*, **, ***).
